# Cross-talk between the ER pathway and the lncRNA MAFG-AS1/miR-339-5p/ CDK2 axis promotes progression of ER+ breast cancer and confers tamoxifen resistance

**DOI:** 10.18632/aging.103966

**Published:** 2020-10-24

**Authors:** Jing Feng, Ti Wen, Zhi Li, Liang Feng, Lu Zhou, Zichang Yang, Lu Xu, Sha Shi, Kezuo Hou, Jiming Shen, Xu Han, Yuee Teng

**Affiliations:** 1 Department of Medical Oncology, The First Hospital of China Medical University, Shenyang 110001, China; 2 Key Laboratory of Anticancer Drugs and Biotherapy of Liaoning Province, The First Hospital of China Medical University, Shenyang 110001, China; 3 Liaoning Province Clinical Research Center for Cancer, Shenyang 110001, China; 4 Department of Breast Surgery, The First Hospital of China Medical University, Shenyang 110001, China

**Keywords:** breast cancer, lncRNA, MAFG-AS1, ceRNA, cell cycle, tamoxifen resistance

## Abstract

Hormone receptor-positive breast cancer accounts for around 75% of breast cancers. The estrogen receptor pathway promotes tumor progression and endocrine resistance. Recently, the cross-talk between the ER signaling pathway and cell cycle regulation has been identified. It is necessary to determine the underlying molecular mechanisms involved in the ER signaling pathway and find new target genes for prognosis and drug resistance in ER+ breast cancer. In this study, lncRNA MAFG-AS1 was shown to be up-regulated and associated with poor prognosis in ER+ breast cancer. Functionally, down-regulation of MAFG-AS1 could inhibit cell proliferation and promote apoptosis. In addition, MAFG-AS1 which contained an estrogen-responsive element could promote CDK2 expression by sponging miR-339-5p. Subsequently, MAFG-AS1 and CDK2 were found to be up-regulated in tamoxifen-resistant MCF-7 cells. Cross-talk between the ER signaling pathway and cell cycle conducted by MAFG-AS1 and CDK2 could promote tamoxifen resistance. In conclusion, our study indicated that estrogen-responsive lncRNA MAFG-AS1 up-regulated CDK2 by sponging miR-339-5p, which promoted ER+ breast cancer proliferation. Cross-talk between the ER signaling pathway and cell cycle suggested that lncRNA MAFG-AS1 is a potential biomarker and therapeutic target in ER+ breast cancer. CDK2 inhibitors may be applied to endocrine resistance therapy.

## INTRODUCTION

Breast cancer is the most common malignancy in women around the world [1, 2]. Estrogen receptor (ER)-positive breast cancer accounts for around two-thirds of all breast cancers which are considered to be estrogen responsive [3]. The estrogen signaling pathway is mainly mediated through ER-alpha which promotes breast carcinogenesis [4]. Compared to other subtypes of breast cancers, patients with ER-positive (ER+) breast cancer have favorable prognosis [5–7]. However, they account for a large proportion of breast cancer deaths as a result of primary or secondary endocrine resistance. Eventually, one third of patients respond to tamoxifen adjuvant therapy upon relapse for breast cancer due to endocrine resistance [8, 9]. In addition, although these patients have undergone postoperative adjuvant therapy, the proportion of tumor recurrence remains high within 5-10 years after surgery [10]. Therefore, there is an urgent need to develop new biomarkers and identify effective targets for disease prognosis and treatment, and to better predict the efficacy of endocrine therapy in ER+ breast cancer.

Long non-coding RNAs (lncRNAs) are a type of RNA with a transcript length of more than 200 nt. LncRNAs do not encode proteins, but regulate the expression of genes in the form of RNA at various levels (epigenetic regulation, transcriptional regulation, post-transcriptional regulation) [11]. Although most functions of lncRNAs remain unclear, studies have shown that lncRNAs play a regulatory role in breast cancer through various mechanisms, acting as oncogenes or tumor suppressor genes [12–15]. Additionally, using high-throughput sequencing from patients with breast cancer, the expression of lncRNAs is highly subtype-specific [16]. ER-alpha activated by estrogen can bind to lncRNA promoters acting as a transcription factor [17].

Activation of the ER pathway is crucial for cell proliferation, differentiation, and programmed cell death [18]. Cross-talk between the intra- and extracellular pathways and ER suggested that endocrine resistance occurs in multiple levels involving the cell cycle and the PI3K/AKT/mTOR pathway [19]. In this cross-talk, lncRNAs increase endocrine resistance and the specific mechanism of estrogen-induced lncRNA in the progression of cell cycle and tamoxifen resistance is still unclear. Estrogen-induced lncRNA DSCAM-AS1 is reported to be highly and specifically expressed in luminal-subtype breast cancer and mediates the development of ER+ breast cancer, and tamoxifen resistance through the interacting protein, hnRNPL [20]. LncRNA LOL acts as a natural sponge for let-7 to regulate tumor progression and tamoxifen resistance in luminal breast cancer [21]. Thus, there is an urgent need to elucidate the regulatory mechanisms of lncRNAs involved in ER+ breast cancer and explore the underlying molecular mechanisms involved in the ER signaling pathway.

Previous studies had confirmed that lncRNA MAFG-AS1 promoted tumor progression in hepatocellular carcinoma [22], lung cancer [23], colorectal cancer [24], and breast cancer [25]. As for breast cancer, research has mainly focused on the triple-negative sub-type, the more aggressive of its kind, but the cause of the higher expression of MAFG-AS1 in luminal subtype, the less aggressive sub-type is unknown. In this study, estrogen-regulated MAFG-AS1, which contained an ER promoter binding region, was shown to be up-regulated in ER+ breast cancer to a greater extent than in ER-negative breast cancer and indicated poor prognosis. It targeted CDK2 by sponging miR-339-5p to promote cell proliferation. We also found that cross-talk between the ER signaling pathway and cell cycle regulation conducted by MAFG-AS1 and CDK2 enhanced tamoxifen resistance. Further investigations are necessary to ascertain the role of MAFG-AS1 in promoting luminal breast cancer progression and tamoxifen resistance. These studies may facilitate the development of new therapeutic targets for breast cancer intervention.

## RESULTS

### Identification of lncRNAs associated with poor survival in ER+ breast cancer

To identify markers of poor prognosis in ER**+** breast cancer, we screened six breast cancer datasets from the GEO database (http://www.ncbi.nlm.nih.gov/geo) for a survival-based meta-analysis. Firstly, the number of patients in each dataset which included survival data was greater than 100. Gene expression levels were detected by using the Affymetrix HGU133PLUS2 chip. Next, a meta-analysis based on overall survival (OS) and recurrence-free survival (RFS) (or distance metastasis-free survival (DMFS)) was respectively conducted to screen for genes by the survcomp function package. After taking the intersection of genes from two meta-analyses, the top 1000 genes were selected from the average of ranking. Finally, the genes which contained 37 lncRNAs were annotated (Supplementary Table 1).

**Figure 1:**
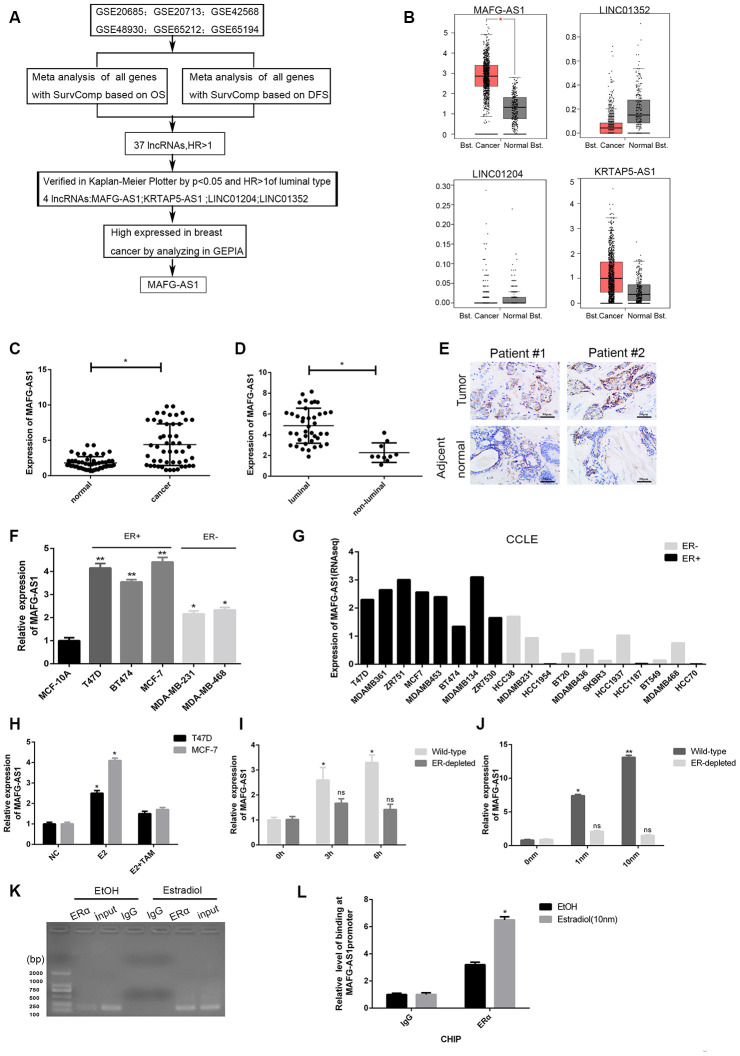
**Identification of lncRNAs associated with poor survival and related to ER+ breast cancer.** (**A**) The flow chart of identifying target lncRNA using bioinformatics methods. (**B**) The expression level plots of four lncRNAs in cancer and normal tissues of breast cancer recorded in GEPIAE (http://gepia.cancer-pku.cn/index.html). Only the expression of MAFG-AS1 in breast cancer (n=1085) and adjacent normal tissues (n=291) is significantly different (p<0.05). (**C**) Expression of MAFG-AS1 in 50 breast tumors compared with para-carcinoma normal tissues. (**D**) Expression of MAFG-AS1 was higher in luminal breast cancer (n=40) than in non-luminal breast cancer (n=10)(*p<0.05). (**E**) Representative ISH (in situ hybridization) detection images of MAFG-AS1 expression in matched normal and primary tumors from two ER positive patients are shown. Scale bars, 50um. (**F**) Relative MAFG-AS1 expression in 5 breast cancer cell lines and 1 normal breast cell line. Error bars represent mean ±SD for triplicate experiments, *p <0.05,**p<0.01. (**G**) MAFG-AS1 was specifically highly expressed in ER+ breast cancer cell lines, as determined by analyzing the RNA-seq data from CCLE (Cancer Cell Line Encyclopedia). (**H**) qPCR expression of MAFG-AS1 8 h following addition of DMSO vehicle, 10 nM estrogen with or without tamoxifen (1uM) in MCF-7 and T47D cell lines. (**I**) The expression of MAFG-AS1 in Wild-type and ERα-depleted T47D cells by different time same concentration. (**J**) qRT-PCR analysis of the expression of MAFG-AS1 in Wild-type and ERα-depleted T47D cells by same time different concentrations. (**K**–**L**) Gel imaging and qPCR-based ChIP analysis of the MAFG-AS1 promoter following ChIP for ERα following 12hr estradiol or DMSO vehicle stimulation. Expression normalized to IgG pulldown. Error bars represent mean±SD.

To further select lncRNAs related to OS or RFS, 37 lncRNAs were subjected to OS and RFS survival analysis in Kaplan-Meier Plotters (https://www.kmplot.com). Four highly expressed lncRNAs indicated poor survival only in luminal breast cancer (P < 0.05 and HR > 1) (Supplementary Figure 1A). We then evaluated the expression levels of the four lncRNAs in GEPIA [26] which were based on TCGA and GTEx data. The entire screening process is shown in Figure 1A. Only lncRNA-MAFG-AS1 was specifically highly expressed in breast cancer (Figure 1B). The survival curves in Kaplan-Meier Plotter show that high MAFG-AS1 expression indicated poor OS for luminal B breast cancer (Supplementary Figure 1B). By AE (any event)-free survival analysis of ER+/HER2-type breast cancer in bc-GenExMiner v4.3 [27], high MAFG-AS1 expression was found to be significantly associated with poor survival (Supplementary Figure 1C). In addition, we found that the PhyloCSF score of MAFG-AS1 was -27.5584 and there were no Bazzini small ORFs in the LNCipedia database (Version 5.2), which indicated that MAFG-AS1 was a non-coding RNA. Based on these, we selected the lncRNA MAFG-AS1 for further investigation which was discovered previously using other tools but same conclusion was reached such that it may be important in breast cancer.

### LncRNA MAFG-AS1 was highly expressed in ER+ breast cancer

For further verification, 50 paired freshly frozen breast cancer tissues and matched adjacent normal breast tissues were collected. qRT-PCR results showed the expression level of MAFG-AS1 was significantly higher in breast cancer tissues than that in adjacent normal breast tissues (Figure 1C, p < 0.05). According to the median expression value of MAFG-AS1 (m_0.5_ = 4.879), the breast cancer tissue samples were divided into high (n = 26) and low (n = 24) expression groups. The expression of MAFG-AS1 was significantly associated with tumor size (p = 0.025) and ki-67 (p = 0.012), but not with other clinicopathological parameters of breast cancer (Table 1). Moreover, the expression of MAFG-AS1 in luminal type breast cancer was higher than in non-luminal type breast cancer (Figure 1D, p < 0.05). In addition, we used *in situ* hybridization (ISH) to detect the MAFG-AS1 expression of two paired breast cancer patients. As shown in Figure 1E, breast cancer specimens exhibited higher MAFG-AS1 expression than adjacent normal tissues, with staining primarily observed in the cell cytoplasm.

**Table 1:** The correlation between the clinicopathological features and expression of MAFG-AS1(n = 50).

**MAFG-AS1 expression**			
**variable**	**Low no. (%)**	**High no. (%)**	**P-value**
Age (y)			.551
≤50	9(37.50)	15(57.69)	
>50	15(62.50)	11(42.31)	
Size (cm)			.025
≤2	15(62.50)	8(30.77)	
>2	9(37.50)	18(69.23)	
Lymph node			.374
-	18(75.00)	16(61.54)	
+	6(25.00)	10(38.46)	
ER			.082
-	6(25.00)	9(34.62)	
+	18(75.00)	17(65.38)	
PR			.598
-	11(45.83)	10(38.46)	
+	13(54.17)	16(61.54)	
HER2			.353
-	15(62.50)	18(69.23)	
+	9(37.50)	8(30.77)	
Ki67 (%)			.012
<14	17(70.83)	7(26.92)	
≥14	7(29.17)	19(73.08)	
Molecular subtype			.177
Luminal A	5(20.83)	4(15.38)	
Luminal B	12(50.00)	19(73.08)	
Her2+	5(20.83)	2(7.69)	
TNBC	2(8.33)	1(3.85)	

Subsequently, qRT-PCR performed in cell lines demonstrated that MAFG-AS1 was highly expressed in all breast cancer cell lines (MCF-7, T47D, BT474, MDA-MB-231, MDA-MB-468) relative to the human immortalized breast epithelium cells (MCF-10A). In particular, MAFG-AS1 was very highly expressed in ER+ breast cancer cell lines (T47D and MCF-7) (Figure 1F). For gene expression of commonly used cell lines in CCLE [28], MAFG-AS1 was generally more highly expressed in eight ER+ lines compared to the eleven ER-negative breast cancer cell lines (Figure 1G). In addition, with different subtypes of breast cancer, bc-GenExMiner v4.3, a database focused on breast cancer, concluded that MAFG-AS1 was relatively highly expressed in luminal A and luminal B breast cancers according to Sorlie's subtype (Supplementary Figure 1D). Similarly, MAFG-AS1 was found to be highly expressed in ER+ and not triple-negative breast cancer (not TNBC) (Supplementary Figure 1E, 1F). These data indicated that the upregulation of MAFG-AS1 might facilitate the progression of ER+ breast cancer progression.

### MAFG-AS1 was estrogen-responsive and directly regulated by ERα

Since MAFG-AS1 was highly expressed in ER+ breast cancer, we explored whether MAFG-AS1 is an estrogen-responsive target gene. Interestingly, MAFG-AS1 expression was markedly induced by E2 treatment in both MCF-7 and T47D cells, which can be reversed by the addition of tamoxifen (Figure 1H). Next, we used wild-type T47D cells deprived of steroid hormones for 3 days [29]. The expression of MAFG-AS1 was induced by estrogen in a dose- and time- dependent manner (Figure 1I, 1J). However, knockdown of the ESR1 gene encoding estrogen receptor 1 (ERα) by RNA interference (Supplementary Figure 1G), attenuated the E2-induced expression level of MAFG-AS1 in T47D cells (Figure 1I, 1J), indicating that MAFG-AS1 expression might be related to ERα expression.

To determine whether the MAFG-AS1 promoter contained an ERα binding region, we predicted the binding map of the promoter region of MAFG-AS1 and ERα in Jaspar (http://jaspar.genereg.net/). As expected, a binding site with the sequence AAAGGTGGCTCTGGCCAC (Supplementary Figure 1H) was identified. In addition, the binding site could also be found in PROMO (http://alggen.lsi.upc.es/cgi-bin/promo_v3/promo/promoinit.cgi?dirDB=TF_8.3) (Supplementary Figure 1I). ESR1 chromatin immunoprecipitation-sequencing (ChIP-seq) in T47D identified ERα binding to the MAFG-AS1 promoter (Supplementary Figure 1J). Furthermore, chromatin immunoprecipitation (ChIP) assay identified ERα binding to the MAFG-AS1 promoter following estrogen stimulation in T47D (IgG as negative control) (Figure 1K, 1L). Taken together, these results suggested that MAFG-AS1 expression was estrogen-responsive and dependent on ERα in luminal breast cancer cells. MAFG-AS1 might be an important target in the development of ER+ breast cancer.

### MAFG-AS1 promoted proliferation of ER+ breast cancer by inducing G1/S cell cycle transition

**Figure 2:**
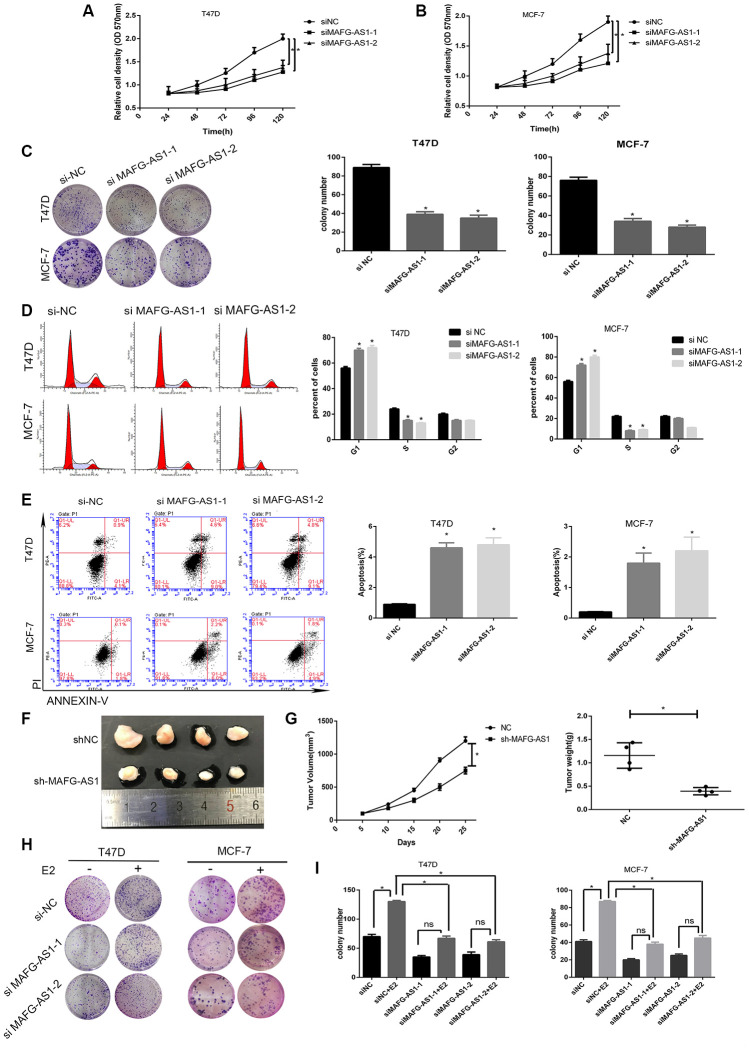
**lncRNA MAFG-AS1 promotes the development of ER+ breast cancer through cell cycle and apoptosis.** (**A**, **B**) Knockdown of MAFG-AS1 significantly decreased cell proliferation compared with si-NC cells in T47D and MCF-7 cell lines by MTT assay. (**C**) Colony formation number of T47D and MCF-7 cells transfected with si-MAFG-AS1 were significantly less than those transfected with si-NC. (**D**) MAFG-AS1 promoted cell cycle from G1 to S phase by flow cytometry. (**E**) MAFG-AS1 promoted antiapoptosis by Annexin-V/FITC double staining on cell apoptosis; the bar graph presents the percentage of apoptotic cells. (**F**) Tumor images of MCF-7-shNC and MCF-7-shMAFG-AS1 cells which were infected with lentiviruses carrying the indicated expression constructs injected into female nude mice pad for in situ tumor formation. (**G**) Tumor volume (left) and weight (right) in the sh-MAFG-AS1 group were significantly lower than those in the sh-NC group. (**H**, **I**) Colony formation of E2 combined with or without knocking down MAFG-AS1. Data are presented as the mean±SD of three independent experiments. *p<0.05.**p<0.01.

To reveal the role of MAFG-AS1 in breast cancer progression, we used MCF-7 and T47D ER+ breast cancer cell lines for *in vitro* experiments. Two MAFG-AS1 specific knockdown sequences (si-MAFG-AS-1 and si-MAFG-AS1-2) were designed and synthesized, and the knockdown efficiency of both cells was detected by qRT-PCR to be > 50% (p < 0.01) (Supplementary Figure 2A, 2B). A significantly reduced proliferation rate was observed in MAFG-AS1-knockdown MCF-7 and T47D cells compared to control cells (Figure 2A, 2B). Moreover, colony formation assays confirmed that the numbers of colonies in the si-MAFG-AS1-1 and si-MAFG-AS1-2 groups were significantly decreased compared to those in the control group (Figure 2C). To further explore whether the observed phenomenon was related to cell cycle, flow cytometry analysis was performed. MAFG-AS1 knockdown arrested the cell cycle at G1 phase in both MCF-7 and T47D cell lines (Figure 2D). Also, the number of apoptotic cells in both cell lines increased after knockdown of MAFG-AS1 (Figure 2E), indicating an anti-apoptotic effect.

To further confirm our *in vitro* findings, stable lentiviral transfected cell lines of MCF-7-shNC and MCF-7-shMAFG-AS1 were constructed, which were then injected into the fat pads of female nude mice for *in situ* tumor formation. The results showed that the shMAFG-AS1 xenograft tumors had a smaller volume and lower weight than that in control group (Figure 2F, 2G). Furthermore, we analyzed the role of MAFG-AS1 in tumor proliferation promoted by estrogen. MTT and colony formation assays indicated that the addition of estrogen could not completely restore the inhibition of proliferation caused by MAFG-AS1 knockdown in both cell lines (Figure 2H, 2I, Supplementary Figure 2C, 2D). The improved growth with estrogen in each case, despite the presence of MAFG-AS1 knockdown, suggests that other mechanisms of estrogen-induced growth remain in place. The above results suggested that MAFG-AS1 promoted proliferation by inducing G1/S cell cycle, and that tumor proliferation promoted by estrogen was partially dependent on MAFG-AS1.

### MAFG-AS1 acts as a competitive endogenous RNA of miR-339-5p

**Figure 3:**
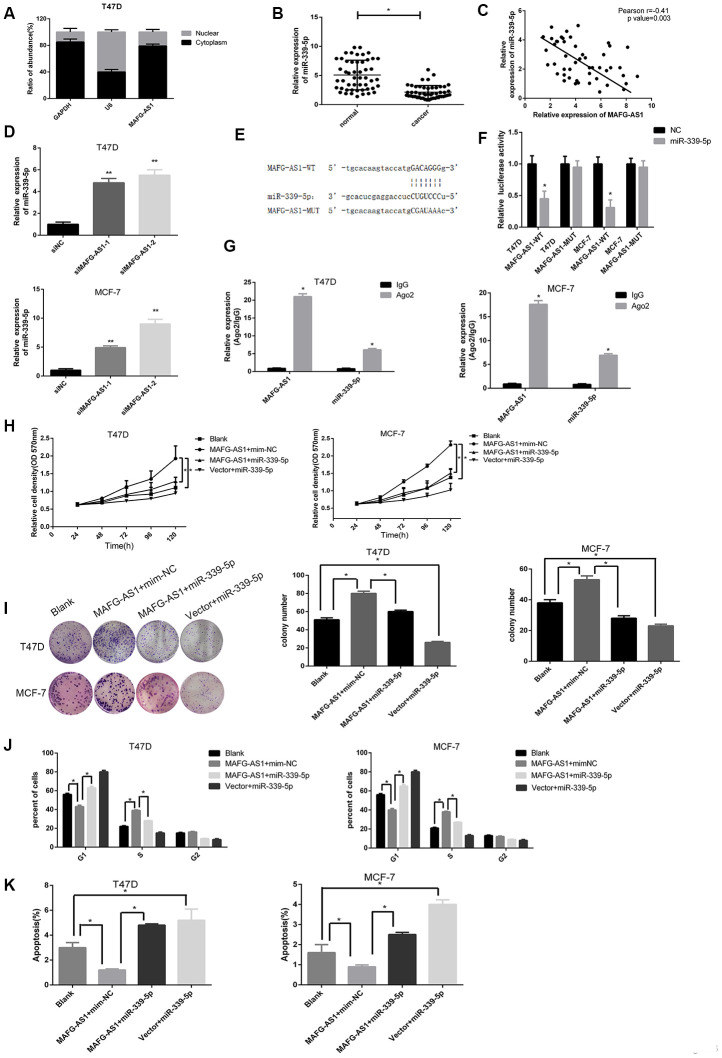
**MAFG-AS1 promotes the progression of ER+ breast cancer through sponging miR-339-5p.** (**A**) The expression of MAFG-AS1 was higher in cytoplasm than nucleus in T47D cells. (**B**) Expression of miR-339-5p in 50 breast tumors compared with para-carcinoma tissues *p<0.05. (**C**) Expression of miR-339-5p and MAFG-AS1 from 50 breast tumors were negatively correlated. (**D**) miR-339-5p expression after siMAFG-AS1-1, siMAFG-AS1-2 or NC-siRNA transfection in T47D and MCF-7 cells.18s was used as an internal control. (**E**) Bioinformatic analysis revealed the presence of complementary binding sites for miR-339-5p in MAFG-AS1. (**F**) Luciferase activity in T47D/MCF-7 cells cotransfected with miR-339-5p and luciferase reporters containing pmirGLO-MAFG-AS1-WT or pmirGLO- MAFG- AS1-MUT. (**G**) The expression of MAFG-AS1 and miR-339-5p after anti-Ago2 RIP were performed in T47D and MCF-7. IgG was used as a negative control. (**H**, **I**) MiR-339-5p overexpression abolished the cell proliferation induced by MAFG-AS1 shown by both MTT assay and colony formation assay. (**J**) Overexpression of MAFG-AS1 cells displayed a significantly low frequency of cells at G1 phase and a high frequency of cells at S phase, while miR-339-5p reversed it. (**K**) MiR-339-5p restored the MAFG-AS1 induced repressing of cell apoptosis.

To elucidate the potential mechanism by which MAFG-AS1 functioned in breast cancer cells, the subcellular localization of MAFG-AS1 by RNA nucleus-cytoplasm separation assay was investigated. In MCF-7 and T47D cell lines, it was shown that that MAFG-AS1 was mainly located in the cytoplasm (Figure 3A, Supplementary Figure 3A). ISH of two primary tumors also showed that MAFG-AS1 was cytoplasmic therein (Figure 1E). Additionally, the lncATLAS (http://lncatlas.crg.eu/) [30] which can predict the subcellular distribution of lncRNAs confirmed that MAFG-AS1 was mainly located in cytoplasm in MCF-7 cells (Supplementary Figure 3B). It is known that cytoplasmic lncRNAs can typically sponge specific microRNAs and act as competitive endogenous RNAs (ceRNAs). MAFG-AS1 is therefore likely to function as a form of ceRNAs. We used an online bioinformatics database (starBasev2.0) [31], the same tool as used in previous studies [25], to predict the potential targets for MAFG-AS1 and found only one target microRNA, hsa-miR-339-5p (Supplementary Table 2), which had previously been reported as a significant tumor suppressor in breast cancer [32].

qPCR data showed that miR-339-5p was down-regulated in 50 paired breast cancer tissues compared to adjacent normal breast tissues, and a negative correlation between MAFG-AS1 and miR-339-5p expression was calculated by Spearman correlation analysis (Figure 3B, 3C). Furthermore, expression of miR-339-5p was up-regulated after MAFG-AS1 knockdown (Figure 3D), whereas overexpression of MAFG-AS1 (Supplementary Figure 3C) exhibited dramatic inhibition of miR-339-5p in MCF-7 and T47D cells (Supplementary Figure 3D). Conversely, overexpression of miR-339-5p could inhibit MAFG-AS1 expression (Supplementary Figure 3E, 3F).

In addition, a binding site of miR-339-5p with MAFG-AS1 was found in RNA22 (https://cm.jefferson.edu/rna22/) [33] (Figure 3E). This site was then mutated for the dual- luciferase reporter assay. Results demonstrated that miR-339-5p overexpression reduced the luciferase activity of the pmir-GLO-MAFG-AS1-WT plasmid, while there was no significant difference in the luciferase activity of the pmirGLO-MAFG-AS1-MUT plasmid in both cell lines (Figure 3F). To investigate whether MAFG-AS1 interacted with miR-339-5p in an Ago2-dependent manner, we performed a RIP assay against Ago2 in T47D and MCF-7 cells. Data showed that a large amount of endogenous MAFG-AS1 and miR-339-5p were precipitated by Ago2 (IgG as a negative control) (Figure 3G), indicating that MAFG-AS1 was involved in sponging and influenced miR-339-5p expression.

To investigate the functional relationship between MAFG-AS1 and miR-339-5p, we performed MTT and colony formation assays, showing that miR-339-5p could attenuate cell proliferation induced by MAFG-AS1 (Figure 3H, 3I). Overexpression of MAFG-AS1 could promote cell cycle transition from G1 to S phase, whilst the addition of miR-339-5p arrested the cell cycle at G1 phase (Figure 3J, Supplementary Figure 3G). The number of apoptotic cells was also decreased by overexpression of MAFG-AS1, which was attenuated by miR-339-5p (Figure 3K, Supplementary Figure 3H). Overall, MAFG-AS1 acted as a competitive endogenous RNA for miR-339-5p in both structure and function.

### CDK2 was a functional target of miR-339-5p and inhibited by miR-339-5p

**Figure 4:**
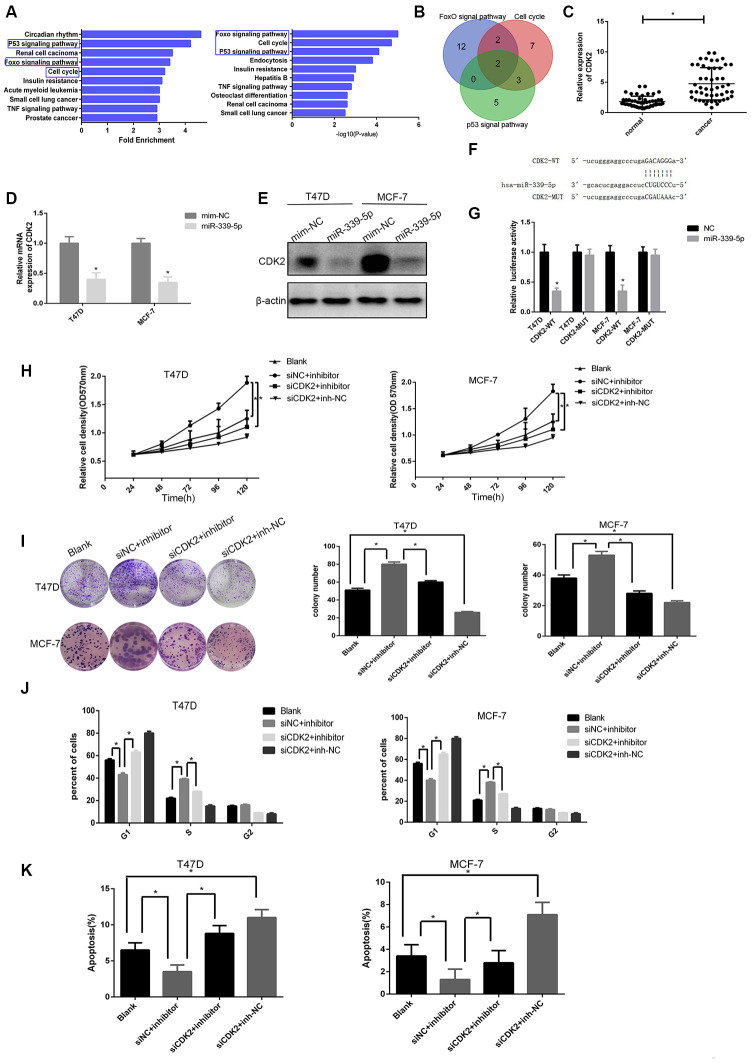
**miR-339-5p inhibits breast cancer through targeting CDK2.** (**A**) Top10 miR-339-5p target gene enrichment pathways according to fold enrichment in descending order and p value in ascending order. (**B**) The intersection of the Venn plot of genes that three pathways contained shown two target genes. (**C**) Expression of CDK2 in 50 breast tumors compared with para-carcinoma tissues *p<0.05. (**D**–**E**) The mRNA and protein levels of CDK2 in T47D and MCF-7 cells after being transfected with miR-339-5p mimics or NC mimics. (**F**) Bioinformatic analysis revealing the presence of complementary binding sites for miR-339-5p with CDK2 in RNA22. (**G**) Luciferase activity cotransfected with miR-339-5p and luciferase reporters containing pmirGLO-CDK2-WT or pmirGLO-CDK2-MUT in T47D/MCF-7 cells. (**H**, **I**) Knockdown of CDK2 abolished the miR-339-5p inhibitor induced cell growth shown by MTT assay and colony formation assay. (**J**–**K**) The cell cycle distribution and apoptosis of T47D and MCF-7 cells.

To explore the target gene of miR-339-5p, bioinformatics analysis was conducted using starBasev2.0 which predicted 704 target genes (Supplementary Table 3). We introduced these genes into DAVID (https://david.ncifcrf.gov/) [34], the pathway enrichment database, which identified pathways according to the fold enrichment in descending order and *P* values in ascending order. We took the top five pathways respectively in order and found a FoxO signaling pathway, cell cycle, and p53 signaling pathway all therein (Figure 4A). Genes contained in the three pathways were further taken together and had two common genes, CDK2 and CCND2, which were closely related to cell proliferation (Figure 4B). We noticed that CDK2 has been reported to be up-regulated and promote the progression of breast cancer [35] and so was selected as a target gene.

Similarly, qRT-PCR data showed that CDK2 was up-regulated in 50 paired breast cancer tissues (Figure 4C). Furthermore, CDK2 mRNA and protein levels were significantly decreased after overexpression of miR-339-5p (Figure 4D, 4E), and miR- 339-5p inhibition significantly increased the expression of CDK2 in MCF-7 and T47D cells (Supplementary Figure 4A, 4B). miR-339-5p had putative CDK2 binding sites in RNA22 (Figure 4F). Dual-luciferase reporter assay results indicated that overexpression of miR-339-5p reduced the luciferase activity of the pmir-GLO-CDK2-WT plasmid, whereas the luciferase activity of the pmirGLO- CDK2- MUT plasmid showed no significant difference in MCF-7 and T47D cells (Figure 4G). These data indicated that miR-339-5p targeted CDK2 and regulated CDK2 expression.

To explore biological function, MTT and colony formation assay showed that inhibition of miR-339-5p promoted the proliferation of MCF-7 and T47D cells which was reversed by CDK2 knockdown (Figure 4H, 4I). In parallel, knockdown of CDK2 arrested the cell cycle at G1 phase. However, miR-339-5p inhibition restored the cell cycle (Figure 4J, Supplementary Figure 4C). Additionally, apoptosis data showed that the inhibition of miR-339-5p counteracted the effect of CDK2 knockdown (Figure 4K, Supplementary Figure 4D). We concluded that miR-339-5p inhibited the proliferation of breast cancer by targeting and inhibiting CDK2.

### MAFG-AS1/miR-339-5p/CDK2 axis regulated proliferation of ER+ breast cancer

**Figure 5:**
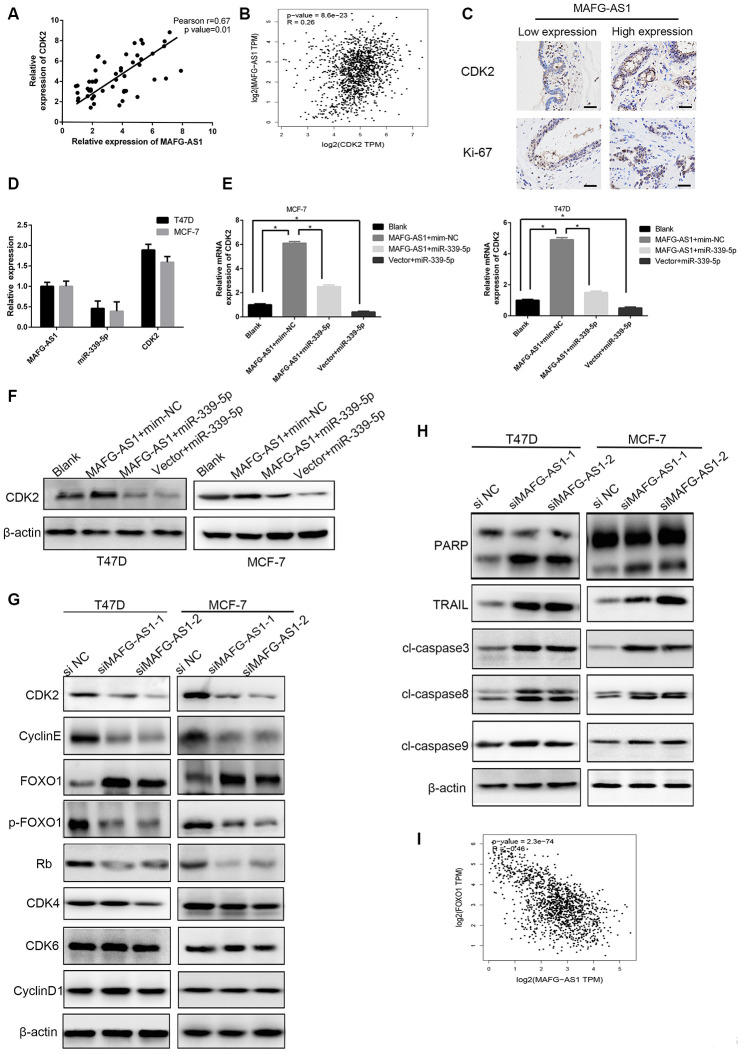
**The MAFG-AS1/miR-339-5p/CDK2 axis regulates cell proliferation.** (**A**) Expression of CDK2 and MAFG-AS1 were positively correlated. p=0.01, r=0.67. (**B**) Correlation between CDK2 and MAFG-AS1 in GEPIA. (**C**) Representative IHC expression of CDK2 and ki-67 shown from two patients with different MAFG-AS1 expressions. (**D**) Relative expression of MAFG-AS1, miR-339-5p and CDK2 in the same cell lines. (**E**) qRT-PCR analysis of the expression of CDK2 with over-expression of MAFG-AS1 and miR-339-5p in T47D and MCF-7 cells. (**F**) Western blot was performed to detect CDK2 expression in T47D and MCF-7 with MAFG-AS1 and miR-339-5p over expression.(**G**) The expression of CDK2, cyclin E, Rb, FOXO1, p-FOXO1, CDK4, CDK6 and CyclinD1 after MAFG-AS1 knockdown compared with NC in T47D and MCF-7 cells. (**H**) The expression of PARP, TRAIL, cl-caspase3, cl-caspase8 and cl-caspase9 after MAFG-AS1 knockdown compared with NC in T47D and MCF-7 cells. (**I**) Correlation between FOXO1 and MAFG-AS1 at mRNA level in GEPIA.

To establish the MAFG-AS1/miR-339-5p/CDK2 axis, a positive correlation between MAFG-AS1 and CDK2 expression was observed in 50 paired breast cancer tissues (Figure 5A), which was the same as the gene-related analysis in GEPIA (r = 0.26, p < 0.0001) (Figure 5B). In addition, we selected two patients with different MAFG-AS1 expressions and the immuno-histochemical staining of breast cancer tissues showed that the expression of CDK2 and ki-67 was consistent with the MAFG-AS1 expression, indicating that MAFG-AS1 was related to proliferation (Figure 5C). The expression of miR-339-5p was almost half that of MAFG-AS1 and CDK2 in MCF-7 and T47D cell lines (Figure 5D). qRT-PCR and Western blotting indicated that overexpression of MAFG-AS1 up-regulated CDK2, while miR-339-5p mimics attenuated the upregulation in MCF-7 and T47D cells (Figure 5E, 5F). Consistently, inhibition of miR-339-5p increased CDK2 expression, which was attenuated by CDK2 knockdown in MCF-7 and T47D cells (Supplementary Figure 4E–4G). These data indicated that MAFG-AS1 functioned as a ceRNA of miR-339-5p to regulate CDK2 in breast cancer cells.

Knowing that CDK2 functions as a significant protein kinase in the control of cell division, we assessed whether the MAFG-AS1/miR-339-5p/CDK2 axis could regulate other cell cycle-related genes. Firstly, mRNA and protein levels of CDK2 were down-regulated after MAFG-AS1 knockdown, and the protein levels of cyclin E and Rb also decreased. However, the cell cycle-associated genes, CDK4, CDK6, and cyclinD1, did not change in MCF-7 and T47D cells (Figure 5G, Supplementary Figure 4H).

We then explored the mechanism related to cell apoptosis. Previous studies have shown that CDK2 interacts with FOXO1 (forkhead transcription factor 1) and phosphorylates it to p-FOXO1, which is inactive. Importantly, activated CDK2 inhibits the pro-apoptotic function of FOXO1 and regulates the transcriptional expression of pro-apoptotic genes such as FasL, Bim and TRAIL [36, 37]. To verify the role of MAFG-AS1, we found that FOXO1 up-regulated after knockdown MAFG-AS1, while inactive p-FOXO1 was down-regulated. Apoptosis-related genes including TRAIL, cl-caspase3, cl-caspase8, and PARP were up-regulated, but cl-caspase9 showed no significant difference. We also found that MAFG-AS1 regulated the process of exogenous apoptosis through CDK2 phosphorylating FOXO1 which was negatively correlated with MAFG-AS1 at mRNA level in GEPIA (r = -0.46, p < 0.0001) (Figure 5H, 5I). These data suggested that the MAFG-AS1/miR-339-5p/CDK2 axis regulated proliferation in ER+ breast cancer.

### MAFG-AS1 and CDK2 promoted tamoxifen resistance

**Figure 6:**
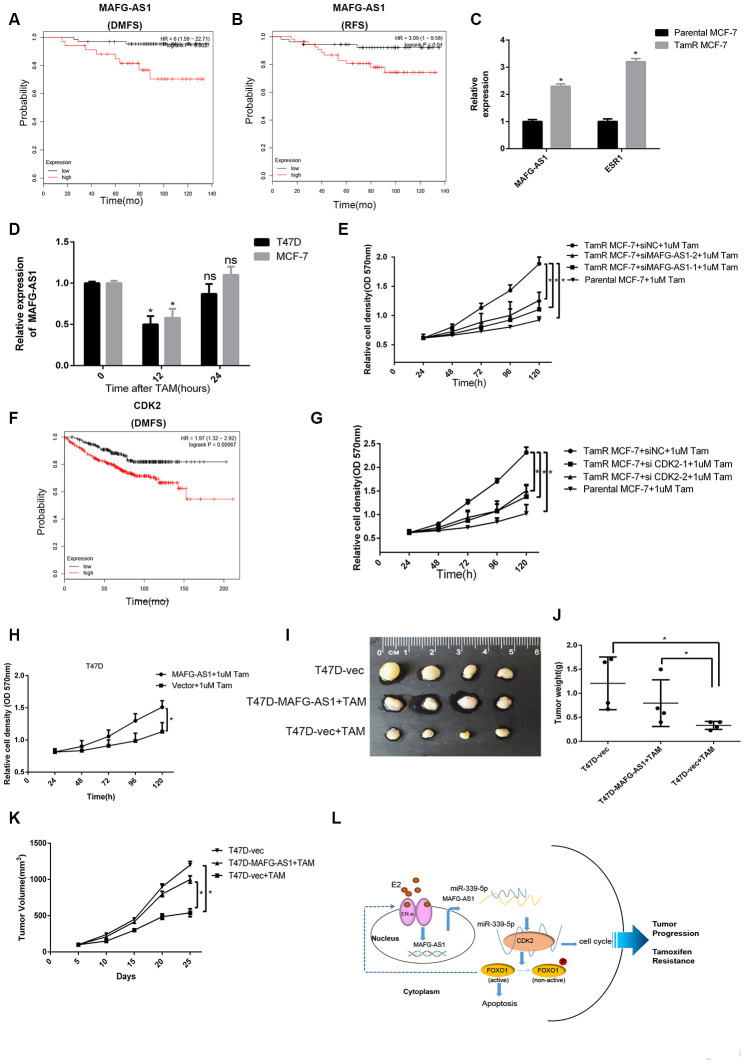
**lncRNA MAFG-AS1 and CDK2 promote tamoxifen resistance.** (**A**, **B**) Kaplan-Meier plotter survival analysis of RFS (HR = 3.09, p = 0.04) and DMFS (HR = 6, p = 0.0027) with the patients treated with adjuvant tamoxifen monotherapy (exclude all chemotherapy). Samples were stratified into “high” and “low” MAFG- AS1 expression based on median cutoff value in each dataset. (**C**) qRT-PCR analysis of the expression of MAFG-AS1 and ESR1 in parental MCF-7 and TamR MCF-7. (**D**) qRT-PCR analysis of the expression of MAFG-AS1 in T47D and MCF-7 cells with the duration of tamoxifen. (**E**) Proliferation assay in parental MCF-7 cells and in TamR MCF-7 cells following siRNA-mediated knockdown of MAFG-AS1 via two independent siRNAs. (**F**) Kaplan-Meier plotter survival analysis of DMFS (HR = 1.97, p=0.00067) with the patients treated with adjuvant tamoxifen monotherapy by different CDK2 expression. (**G**) Proliferation assay in parental MCF7 cells and in TamR-MCF7 cells following siRNA-mediated knockdown of CDK2 via two independent siRNAs. (**H**) MTT assay performed for T47D cells overexpressing vector control and MAFG-AS1 with 1uM tamoxifen. (**I**–**K**) Tumor volume and weight confirmed the truth of MAFG-AS1 promotes tamoxifen resistance *in vivo* experiment. (**L**) Possible molecular mechanisms of the MAFG-AS1/ miR-339-5p/CDK2 axis and positive feedback loop in luminal breast cancer.

Many ER+ breast cancer patients eventually develop resistance to endocrine therapy with clinical recurrence and metastasis [38]. Considering that MAFG-AS1 is highly expressed in luminal breast cancer with poor prognosis, we hypothesized that it might be related to tamoxifen resistance. Firstly, we selected patients who had adjuvant tamoxifen monotherapy in Kaplan-Meier Plotter, and found that patients with high MAFG-AS1 expression had poor RFS and DMFS (Figure 6A, 6B). MAFG-AS1 was likely to be associated with tamoxifen resistance.

We used the stable tamoxifen-resistant TamR-MCF-7 and parental MCF-7 cells for mechanistic investigation. qRT-PCR analysis indicated that MAFG-AS1 was expressed at a very high level in MCF-7 cells, but it was up-regulated significantly in TamR-MCF-7. The level of ER was also increased which might result from the compensatory up-regulation of the anti- estrogen action of tamoxifen (Figure 6C). In addition, short-term tamoxifen treatment of parental MCF-7 cells temporarily decreased MAFG-AS1 levels at 12 hours, which returned to pre-treatment levels after 24 hours (Figure 6D). T47D cells over-expressing MAFG-AS1 also exhibited a proliferative advantage when grown in estrogen-deprived medium (Supplementary Figure 5A). This indicated that some factors, except estrogen, induce MAFG-AS1 expression, which is independent on estrogen in ER+ breast cancer.

To determine whether upregulation of MAFG-AS1 in TamR-MCF-7 cells has a functional impact, we studied the proliferative capacity of these cells after knockdown of MAFG-AS1. The knockdown level of MAFG-AS1 was similar to the level of parental MCF-7 cells (Supplementary Figure 5B). When cultured in tamoxifen, knockdown of MAFG-AS1 in TamR-MCF7 cells resulted in a reduced proliferation advantage which was similar to parental MCF-7 cells (Figure 6E). Similarly, tamoxifen monotherapy patients with high CDK2 expression had poor DMFS in Kaplan-Meier Plotter (Figure 6F). Knockdown of CDK2 led to a similar reduction of proliferative capacity in TamR-MCF7 cells (Figure 6G, Supplementary Figure 5C). These data suggested that both MAFG-AS1 and CDK2 enhanced tamoxifen resistance.

Next, the ability of MAFG-AS1 to increase tamoxifen resistance in native T47D cells was investigated by MAFG-AS1 overexpression. As expected, MAFG-AS1 overexpression exhibited proliferative advantage more than negative control vectors (Figure 6H). More importantly, we constructed T47D-vector and T47D-MAFG-AS1 lentiviral stable transfected cell lines which were injected into the fat pads of nude mice for *in situ* tumor formation. When the tumor reached a volume of about 100 mm^3^, mice were given tamoxifen treatment. Compared to the group of T47D-vector cells without tamoxifen treatment, the tumor volume and weight of the T47D-MAFG-AS1+TAM group were similar but significantly greater than the T47D-vector+TAM group (Figure 6I–6K). These data indicated that MAFG-AS1 and CDK2 promoted tamoxifen resistance in an estrogen-independent manner in ER+ breast cancer.

## DISCUSSION

ER+ breast cancer is driven by ERα and accounts for 70% of breast cancer death. Tumor recurrence remains high 5-10 years after surgery. During this period, the ER signaling pathway promotes the tumor progression therein. Tamoxifen, the anti- estrogen therapy, has become a classic treatment for ER+ breast cancer and reduces one-third of total mortality in advanced breast cancer. However, resistance to tamoxifen therapy is a major factor which significantly influences survival outcomes for ER+ breast cancer.

Cross-talk between the ER signaling pathways and cell cycle has been identified in endocrine resistance. Logically, CDK4/6 inhibitors have entered into the clinic. CDK2, another vital G1/S phase regulator, has been reported as the cause of resistance to CDK4/6 inhibitors [39]. However, there is little evidence of endocrine resistance between CDK2 and ER+ breast cancer. LncRNAs which have mRNA-like structure cannot encode proteins and most of their functions are unknown. Although lncRNAs promote the development of cancer, a few lncRNAs are associated with the prognosis of ER+ breast cancer and may be potential therapeutic targets. In this study, we found that lncRNA MAFG-AS1 was up-regulated and induced by estrogen, which indicates poor prognosis in ER+ breast cancer. As a clinical prognostic factor in luminal breast cancer patients, MAFG-AS1 could up-regulate CDK2 by sponging miR-339-5p and accelerating the G1/S phase transition. Consequently, cell proliferation and colony formation of tumor cells were promoted. Furthermore, phosphorylation of the transcription factor FOXO1, which was regulated by CDK2, inhibited the activation of apoptosis-related pathways (Figure 6L). We demonstrated that ERα could regulate MAFG-AS1 expression as a transcription factor. Interestingly, ERα was also down-regulated with MAFG-AS1 knockdown (Supplementary Figure 5D, 5E). Thus, MAFG-AS1 and ERα may form a positive feedback loop, implying that they can promote each other to a high level of expression in ER+ breast cancer. It has been reported that FOXO1 can act as a transcription factor for ERα in breast cancer [40, 41], which may contribute to positive feedback formation. This may also explain the mechanism of tamoxifen resistance induced by cross-talk between the cell cycle and ER signaling pathway.

Previous studies on MAFG-AS1 in breast cancer were associated with invasion and metastasis. Li et al. found that the ceRNA network of MAFG-AS1/miR-339-5p/MMP15 promoted invasion and metastasis of breast cancer [25]. This mainly proves the aggressiveness of the mechanism of ER-negative breast cancer. In this study, ER+ breast cancer which was identified to be less aggressive than ER-negative breast cancer was analyzed due to the difference in mechanisms and biological functions of various subtypes of breast cancers, which warrant various treatment options. We discovered that the tumor proliferation function of MAFG-AS1 and miR-339-5p might regulate cell cycle transition. Amongst the regulatory pathways, ERα and CDK2 were proliferation-related indicators, which fully verified the function of MAFG-AS1 in ER+ breast cancer. Further evidence also supports the proliferation mechanism of miR-339-5p during ER+ breast cancer progression.

At present, treatment targeting lncRNA is in its infancy. A more advanced method targeting lncRNA involves antisense oligonucleotides (ASOs), which function more effectively than small interfering RNAs (siRNAs) *in vitro* and *in vivo* [42]. Several ASOs have entered into clinical trials. In future studies, we will apply ASOs targeting MAFG- AS1 as a potential therapeutic target and research their clinical feasibility. The ASOs targeting MAFG-AS1 may further prolong the survival of patients.

This study also showed that MAFG-AS1 and CDK2 were associated with tamoxifen resistance, and that patients with high expression of MAFG-AS1 and CDK2 had poor survival. MAFG-AS1 and CDK2 were likely to be targets for the treatment of tamoxifen resistance. Endocrine therapy resistance, such as tamoxifen resistance, is a major challenge for ER+ breast cancer. It is known that mutations of ESR1 enhance endocrine resistance and the downstream mechanism have an intact Rb axis, which in turn can promote the G1/S phase transition. The transformation of G1/S phase has two pairs of cyclin-CDK complexes, cyclin D-CDK4/6 and cyclin E-CDK2 [43]. CDK4/6 inhibitors have been evaluated clinically for endocrine resistance [44]. In this study, MAFG-AS1 targets CDK2. It is not known whether CDK2 inhibitors can reverse tamoxifen resistance conducted by an ERα- MAFG-AS1-CDK2-ERα positive feedback loop. Recent studies have shown that resistance to CDK4/6 inhibitors may be mediated by cyclinE-CDK2 bypass activation. Thus, CDK2 inhibitors are critical, and it may possibly reverse tamoxifen resistance by combining two kinds of inhibitors or depending on MAFG-AS1 expression to select cell cycle inhibitors.

Finally, we identified the clinical significance of MAFG-AS1 in luminal breast cancer. High MAFG-AS1 expression indicated poor survival. ASOs targeting MAFG-AS1 and CDK2 inhibitors may provide a promising approach to inhibit luminal breast cancer progression and tamoxifen resistance. Cross-talk between the ER signaling pathway and cell cycle was conducted by MAFG-AS1 and CDK2, but not CDK4/6. This provides a new idea for endocrine resistance. Through the investigation of MAFG-AS1, the mechanism of ER+ breast cancer progression was highlighted and further investigation of the other cross-talk with the ER signaling pathway may produce greater discovery of ER-mediated tumorigenesis.

In summary, our study demonstrated that estrogen-responsive MAFG-AS1 functions as an oncogenic lncRNA during ER+ breast cancer progression. Our data showed a novel ceRNA regulatory pathway in which MAFG-AS1 up-regulates CDK2 expression by sponging miR-339-5p. In addition, MAFG-AS1 and CDK2 confer tamoxifen resistance and indicate poor survival. Cross-talk between MAFG-AS1/miR-339-5p/CDK2 axis and ER signaling pathway is potentially a novel direction for endocrine resistance therapy. MAFG-AS1 and CDK2 may become attractive therapeutic targets in ER+ breast cancer.

## MATERIALS AND METHODS

### Patients and tissue specimens

For this study, fifty paired of tissue specimens and matched normal tissue samples obtained from the breast cancer patients underwent operation at the First Affiliated Hospital of China Medical University from December 2017 to August 2019. All patients received no chemotherapy or radiotherapy before enrollment. Tissues were collected within a half hour following surgery, frozen in liquid nitrogen overnight, and kept at -80°C until the isolation of total RNA. All patients signed an ethical consent form. This study was approved by the Ethic Committee of the First Affiliated Hospital of China Medical University.

### Cell culture

MCF-7, BT474, T47D and MCF-10A cells used in this study were acquired from ATCC (Manassas, VA, USA). MCF-7, BT474 and T47D express estrogen receptors and respond mitogenically to 17β-oestradiol. MDA-MB-231 and MDA-MB-468 cell lines, which are triple-negative (no HER2/neu amplification and no receptors for progesterone or estrogen) were obtained from Cell Bank of Type Culture Collection of Chinese Academy of Sciences (Shanghai, China). BT474 cells were cultured in RPMI 1640 medium (Hyclone, GE, USA). MCF-7 cells were maintained in Dulbecco's modified Eagle's medium (DMEM; Thermo Fisher Scientific, MA, USA) with 0.01 mg/mL insulin (Solarbio, Beijing, China). T47D cells were maintained in DMEM (Thermo Fisher Scientific, MA, USA). MDA-MB-231and MDA-MB-468 were cultured in L-15 medium using impermeable flasks. Cells were cultured with 10% FBS, 100 U/mL penicillin, and 0.1 mg/mL streptomycin at 37°C in 5% CO_2_. MCF-10A cells were cultured in DMEM/F12 medium with 5% horse serum, 100 ng/mL cholera, 10 ug/mL insulin, 20 ng/mL epidermal growth factor (EGF) toxin and 500 ng/mL hydrocortisone. Specifically, hormone-deprived medium (HD) was obtained from phenol red-free DMEM (ThermoFisher Scientific) supplemented with 5% charcoal-stripped FBS (Biological Industries, 04-201-1B); 17β-estradiol (E2) (Sigma, E2758) and 4-hydroxytamoxifen (T176-10MG) were added in specific experiments; Tamoxifen Resistance (TamR) MCF-7 cells were kindly provided by Professor Guojun Zhang (Department of Oncology, Xiang’an Hospital of Xiamen University, Xiamen, China).

### Vectors and transfections

Vectors were transfected to cells with Lipofectamine 2000 Reagent (Invitrogen). Cells were transfected with siMAFG-AS1, miR-339-5p mimics, inhibitor, or the corresponding negative controls at a final concentration of 50nM (mimics) or 200nM (inhibitor). Cells were collected 48h after transfection. The siMAFG-AS1, siCDK2, siESR1 (ViewSolid Biotech, Beijing, China), miR-339-5p mimics or inhibitor (RiboBio, Guangzhou, China) and corresponding negative control were designed and synthesized. For *in vivo* experiments, MAFG-AS1 was overexpressed and knockdown with a lentiviral system. Lentiviral production and infection were performed following the standard procedure recommended by the company (Shanghai Genechem Co., Ltd.). At 72 h, the virus-infected cells were used for experimental analysis. The siRNA sequences were in Supplementary Table 5.

### Plasmid construction

The sequence corresponding to the wild-type MAFG-AS1(NR_015454) was amplified by PCR and inserted in the reporter plasmid pmirGLO (Promega, WI, USA). The plasmid was named as pmirGLO-MAFG-AS1-WT. For construction of MAFG-AS1 reporter gene plasmids with a mutant miR-339-5p binding site, the QuikChange site-directed mutagenesis kit (Stratagene, CA, USA) was used according to the manufacturer's construction. These mutant plasmids were named as pmirGLO-MAFG-AS1-MUT. Wild type and mutant inserts were confirmed by DNA sequencing.

### Western blotting

Cell samples were solubilized in 1% Triton lysis buffer on ice and quantified according to the Coomassie blue G250 staining technique. After that, all the samples were added 3× sampling buffer and boiled at 95°C for 5min. Then lysates were separated by SDS-polyacrylamide gel electrophoresis (SDS/PAGE) and electronically transferred to nitrocellulose membranes (Millipore, Bedford, Ma, USA). After blocking with 5% skimmed milk in TBST, the blots were probed with the indicated primary antibodies at 4°C overnight and incubated with horseradish-peroxidase-conjugated secondary antibodies as indicated for 30min at room temperature. Finally, the target bands of proteins were detected with enhanced chemiluminescence reagent (SuperSignal Western Pico Chemiluminescent Substrate; Pierce, Rockford, IL, USA).

### RNA isolation and quantitative real-time PCR

Total RNAs of tissues and cells were extracted by TRIzol reagent (Invitrogen, Carlsbad, CA, USA), and the concentration of the total RNA was quantified by measuring the absorbance at 260nm. All reagents for the reverse transcription (RT) were obtained from TaKaRa. The Prime-Script™ RT reagent Kit (Takara, Japan) and the One Step Prime-Script® miRNA cDNA Synthesis Kit (Takara, Japan) were used for mRNA and miRNA RT. qRT-PCR was performed by SYBR Premix Ex Taq II(TaKaRa) and measured in Applied Biosystems® 7500 Real-Time PCR Systems (ThermoFisher, IL, USA). U6 or 18S was used as the internal control. The data were analyzed using 2^-ΔΔCt^ method. The PCR primers used were in Supplementary Table 4.

### Immunohistochemical (IHC) staining

IHC staining was performed according to protocol. Tissue samples were embedded in paraffin. Briefly, after deparaffinized and rehydrated, the samples were treated with 3% H2O2 for 10 minutes to eliminate intrinsic peroxidase activity, and then incubated overnight at 4 °C with primary antibodies. After washing and incubated with the biotinylated secondary antibodies (Dako, Denmark), the sections were visualized with 3, 3’-iaminobenzidine DAB and counterstained with hematoxylin, and then dehydrated and mounted. Expression of CDK2 and ki-67 were detected by immunohistochemical staining. Sections were visualized under a microscope (400× or 200×) (Olympus, Japan). Ki-67 was examined to evaluate cell proliferation.

### In situ hybridization (ISH)

Expression of MAFG-AS1 in BC was detected using biotin-labeled MAFG-AS1 ISH probes (BOSTER, Wuhan, China) for TMA on the basis of the protocol provided by the manufacturer. Briefly, TMA slides were fixed in 4% paraformaldehyde and then incubated with proteinase-K for 20 min at 37 °C. The slides were hybridized with MAFG-AS1 probe (200 nM) for 40 min at 50 °C. The slides were incubated with anti-DIG reagent, and the probe signal was visualized with diaminobenzidine (DAB) solution (BOSTER). Two pathologists evaluated the IHC and ISH scores in a blinded manner. The intensity of MAFG-AS1, CDK2 and Ki-67 staining was scored on a scale of 1–4 as follows: 1 (no staining), 2 (weak staining), 3(moderate staining) and 4 (strong staining). Tissues with scores of 3 and 4 were defined as high expression group, and those with scores of 1 and 2 were classified as exhibiting low expression.

### Cell proliferation assay

Cell proliferation was measured with the 3-(4,5-dimethyl thiazol-2-yl) -2,5-diphenyl tetrazolium bromide (MTT) assay. Cells (5 × 10^3^ cells/well) were seeded in 96-well plates and tested at 570 nm wavelength with a microplate reader. The data represents the mean ± SD of at least nine wells from three independent experiments.

### Colony formation analysis

Cells were plated in 6-well plates (700 cells/well) and maintained in media containing 10% FBS. The medium was replaced every 4 days. After 14 days, remove the medium from the wells, the cells were fixed with methanol and colonies were stained with Giemsa and images were captured. For each treatment group, wells were assessed in triplicate, and experiments were independently repeated three times. Image J software was used to count colonies.

### Flow cytometry

1×10^6^ cells were harvested and fixed overnight at 4 °C in 70% ethanol. After the cells were washed twice with PBS, their DNA was stained with the Cell Cycle Detection Kit (KeyGen, Nanjin, China). The samples were quantified by flow cytometry (Becton Dickinson, NJ, USA) and results were analyzed with Modfit LT software (Verity Software House, Topsham, ME, USA) according to the manufacturer’s instructions. Cell apoptosis was evaluated using the Annexin-V/Propidium Iodide Detection Kit (Key GEN, China).

### RNA immunoprecipitation assay

RIP experiments were performed using Magna RIP RNA-Binding Protein Immunoprecipitation Kit (Millipore, Billerica, MA, USA) according to the manufacturer’s instructions. Cells were lysed by lysis buffer containing protease inhibitor cocktail and RNase inhibitor. Magnetic beads were preincubated with an anti-Ago2 antibody (Abcam, CA, USA) or anti-rabbit IgG (Millipore, MA, USA) for 1h at room temperature, and lysates were immunoprecipitated with beads at 4°C overnight. RNA was purified, reverse transcribed by cDNA Synthesis Kit (TaKaRa, Japan), and then detected by qRT-PCR.

### Dual luciferase reporter gene assays

A firefly luciferase reporter plasmid (pmirGLO-MAFG-AS1-WT or pmirGLO- MAFG-AS1-MUT) and a renilla luciferase vector (pRL-SV40, Promega) plus small RNAs (miR-339-5p mimics or negative control RNAs) were co-transfected into T47D or MCF-7 cells with Lipofectamine®2000 (Invitrogen, CA, USA). Three independent transfection experiments were performed, and each was done in triplicate. Firefly luciferase activities derived from pmirGLO-control-derived plasmids were normalized to renilla luciferase activity from pRL-SV40 using a luciferase assay system (Promega, WI, USA).

### *In vivo* xenograft animal model

Pathogen-free female BALB/c nude mice (4 weeks of age) were purchased from WeiTongLiHua (Beijing, China). Mice were housed in specific pathogen-free conditions, three per-cage, and maintained at constant temperature (22°C) and humidity. For Figure 2F, estrogen pellets (60-day slow release pellet containing 0.72 mg; Innovative Research of America) were implanted subcutaneously at the nape of the neck of female BALB/c nude mouse. After one week, the mice were randomized into two groups of four mice each. To establish xenograft tumors, a breast cancer cell suspension (1 × 10^7^ cells in 0.2 mL of PBS was injected into the mammary fatty pad of each nude mouse. One group was inoculated with MCF-7 cells infected with lentiviral vector control and the second group was inoculated with MCF-7 cells infected with sh-MAFG-AS1 lentivirus [45]. For Figure 6I, 1 × 10^7^ T47D cells suspended in 0.2mL of PBS/Matrigel (1:1) were injected subcutaneously into the mammary fatty pad of female nude mice with the estrogen pellets implanted 7 days prior. When the tumor size reached ~100 mm3, four mice in each group were treated with or without tamoxifen pellets implanted subcutaneously (60-day slow release pellet containing 5 mg; Innovative Research of America). Tumor growth was measured with fine calipers twice each week and the tumor volume was calculated by the formula shown below: V =(L×W^2^)/2. At the end of the experiment, all mice were humanely euthanized and necropsied. Tumor tissues were harvested, rinsed in saline, weighed, and immediately formalin-fixed.

### Chromatin immunoprecipitation

HighCell ChIP kit (Diagenode) was used to perform ChIP assays via the manufacturer’s protocol. Briefly, MCF-7 cells were grown in charcoal-stripped serum media (described above) for 72 h and then stimulated 10nM estradiol for 12 h. Cells were then crosslinked using 1% formaldehyde for 10 min, and crosslinking was quenched for 5min at room temperature using a 1/10 volume of 1.25M glycine. Cells were then lysed and sonicated (Bioruptor, Diagenode) (two runs of 10 cycles, 30 s on-30s off). DNA bound to immunoprecipitated product was isolated (IPure Kit, Diagenode) via overnight incubation with antibody at 4°C. After washing, DNA was purified and submitted to qPCR analysis with primers mapping to the promoter. Data were processed based on the percentage input method according to the kit manual.

### Statistical analysis

Statistical analyses were performed using SPSS (version 23.0, SPSS Inc.) or GraphPad Prism software (version 6.0, USA). Data are presented as the mean±SD, and differences were determined using the Student's t-test. A p value of less than 0.05 was considered statistically significant. All data were presented as the mean ± standard deviation (SD).

### Ethical approval

The research was approved by the Ethics Committee of the First Affiliated Hospital of China Medical University (Shenyang, China). All patients had signed inform consent forms. This study was carried out in strict accordance with the recommendations in the Guide for the Care and Use of Laboratory Animals of the National Institutes of Health. The protocol was approved by the Committee on the Ethics of Animal Experiments of China Medical University.

## Supplementary Material

Supplementary Figures

Supplementary Table 1

Supplementary Table 2

Supplementary Table 3

Supplementary Table 4 and 5
