# Supplementary material for: Cross-talk between the ER pathway and the lncRNA MAFG-AS1/miR-339-5p/ CDK2 axis promotes progression of ER+ breast cancer and confers tamoxifen resistance
Source: Aging (Albany NY). 2020 Oct 24;12(20):20658–83. doi: 10.18632/aging.103966 (PMC7655217; doi:10.18632/aging.103966)
Supplement: Supplementary Figures [file aging-12-103966-s001..pdf]

SUPPLEMENTARY FIGURES

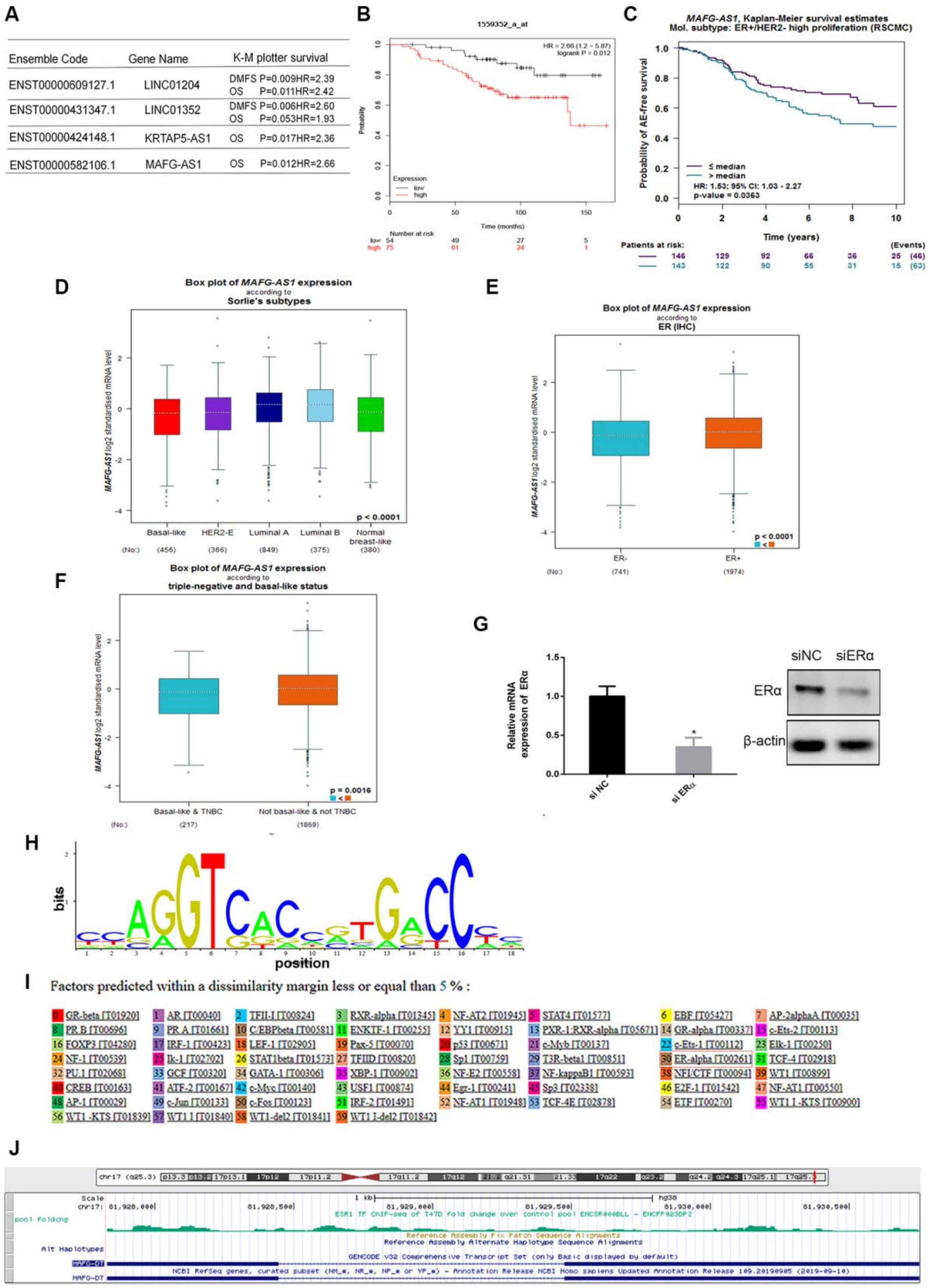

Supplementary Figure 1. MAFG-AS1 is highly expressed in ER+ breast cancer and related to ERα. (**A**) List of four lncRNAs with poor survival in ER positive breast cancer by Kaplan-Meier Plotter. p<0.05, HR>1. (**B**) The K-M plotter survival analysis of OS in luminal B breast cancer which were stratified into “high” and “low” MAFG-AS1 expression based on median cutoff value. (**C**) AE (any event)-free survival analysis of ER+/HER2-type breast cancer in bc-GenExMiner v4.3, found that MAFG-AS1 was significantly associated with poor survival. (**D**) The expression of MAFG-AS1 in breast cancer of different subtypes. LuminalA> Basal-like, LuminalB> Basal-like, LuminalA> normal, LuminalB> normal, LuminalA> HER2, LuminalB> HER2 by Dunnett-Tukey-Kramer's test in Breast Cancer Gene-Expression Miner v4.3

(p<0.05). (**E**) The expression of MAFG-AS1 in ER-positive (n=1974) and ER-negative (n=741) breast cancer, which was analyzed by ER IHC in samples, p<0.0001. (**F**) The expression of MAFG-AS1 in TNBC (n=217) and non-TNBC (n=1869) breast cancer, p=0.0016 from Breast Cancer Gene-Expression Miner v4.3. (**G**) The mRNA and protein expression of ERα after knockdown of ERα. (**H**) Motif binding diagram between ERα and MAFG-AS1. (**I**) Transcription factors predicted by the PROMO bind to the MAFG-AS1 promoter region. (**J**) ESR1 CHIP-seq of T47D compared with the sequence of MAFG-AS1.

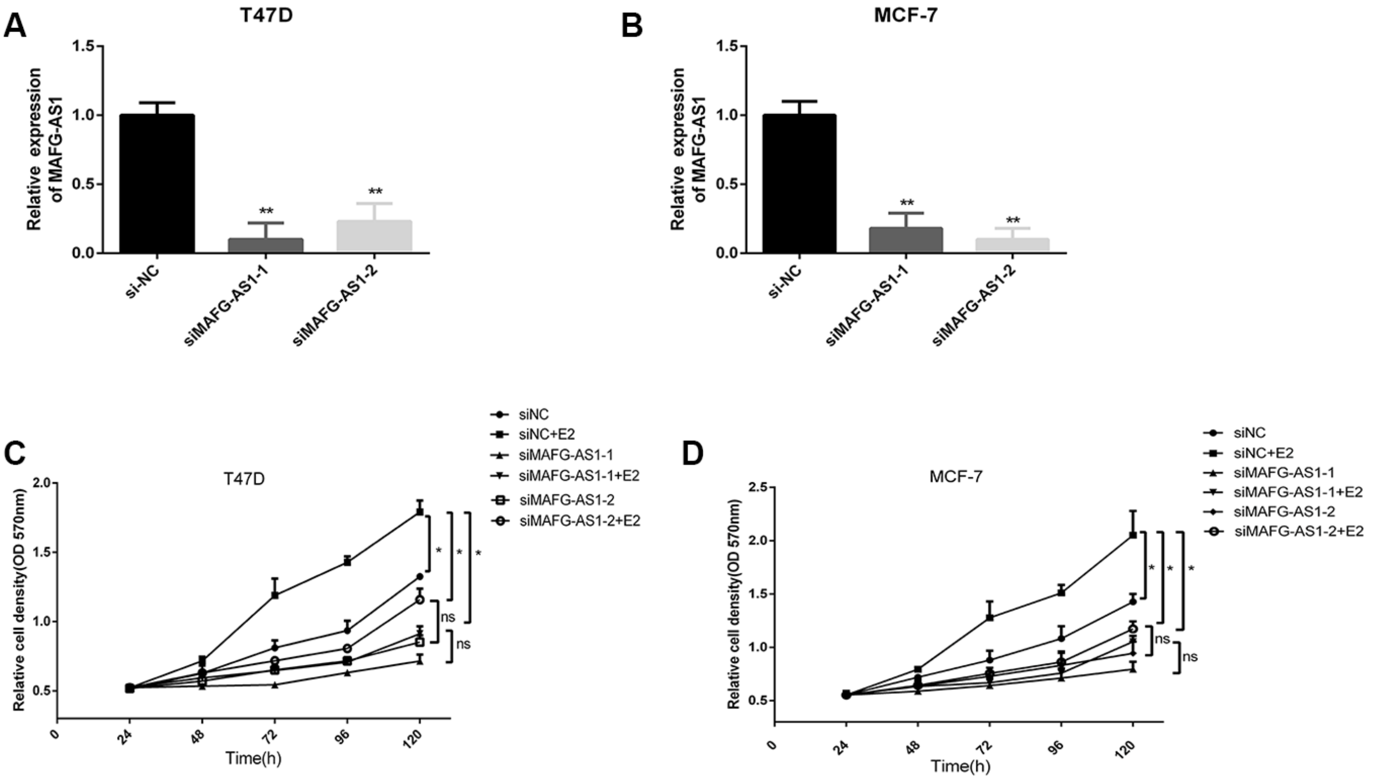

Supplementary Figure 2. MAFG-AS1 knockdown efficiency and MTT assay. (**A**, **B**) Knockdown efficiency of MAFG-AS1 in MCF-7 and T47D cells. (**C**, **D**) MTT assay of E2 combined with or without knocking down MAFG-AS1. Data are presented as the mean ± SD of three independent experiments. *p<0.05.

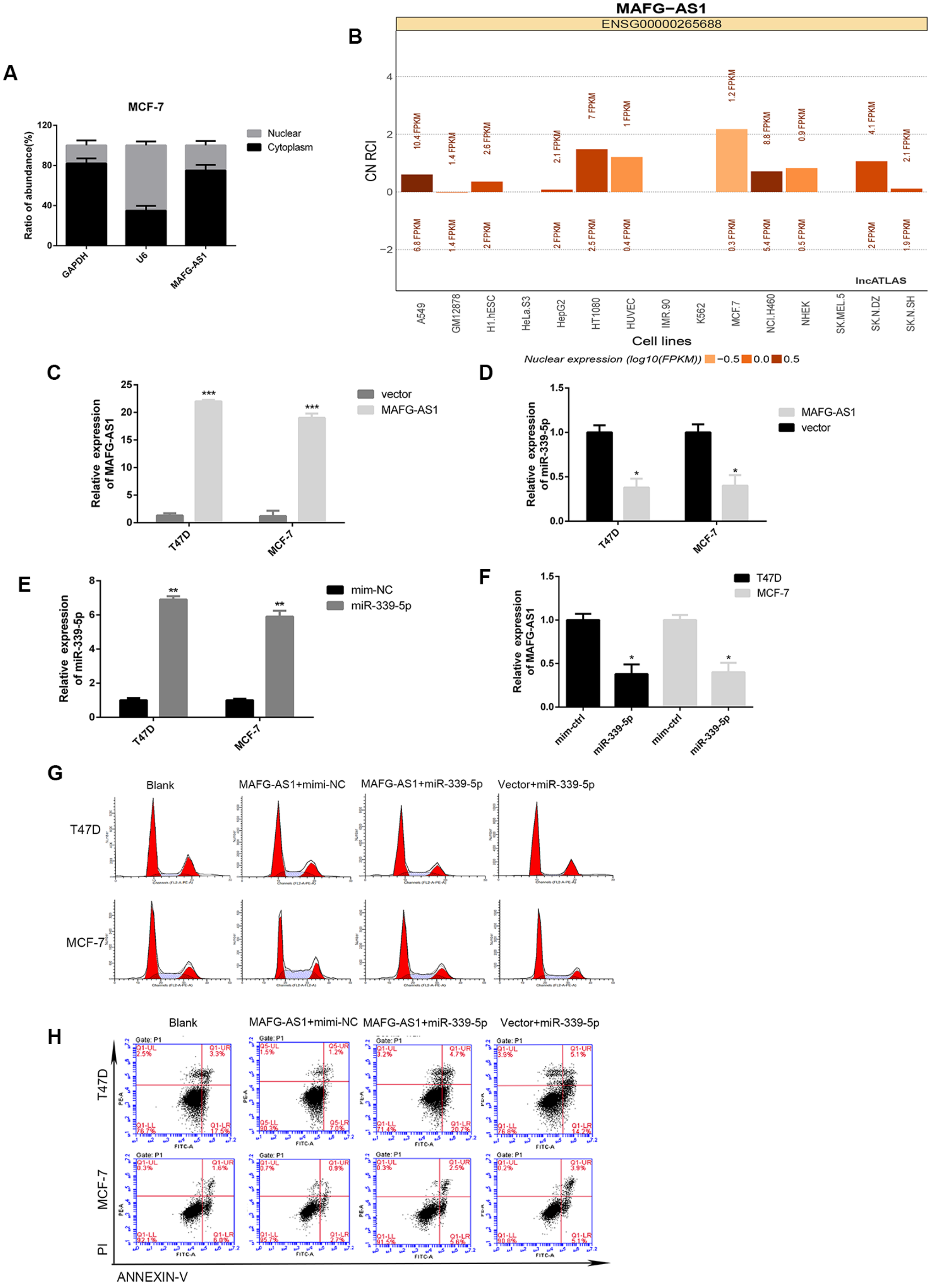

Supplementary Figure 3. Correlation between MAFG-AS1 and miR-339-5p. (**A**) The expression of MAFG-AS1 was higher in cytoplasm than nucleus in MCF-7 cells. (**B**) Nucleus and cytoplasm expression of MAFG-AS1 in different cells obtained from lncATLAS. (**C**) Overexpression efficiency of MAFG-AS1 in MCF-7 and T47D cells. (**D**) The expression of miR-339-5p after OE-MAFG-AS1 or OE-NC transfection in T47D and MCF-7 cells. (**E**) Overexpression efficiency of miR-339-5p in MCF-7 and T47D cells. (**F**) The expression levels of MAFG-AS1 after being transfected with miR-339-5p mimics or NC mimics in T47D and MCF-7 cells. 18 S was used as an internal control. (**G**) Overexpression of MAFG-AS1 cells displayed a significantly low frequency of cells at G1 phase and a high frequency of cells at S phase, while miR-339-5p reversed it. (**H**) MiR-339-5p restored the MAFG-AS1 induced repressing of cell apoptosis.

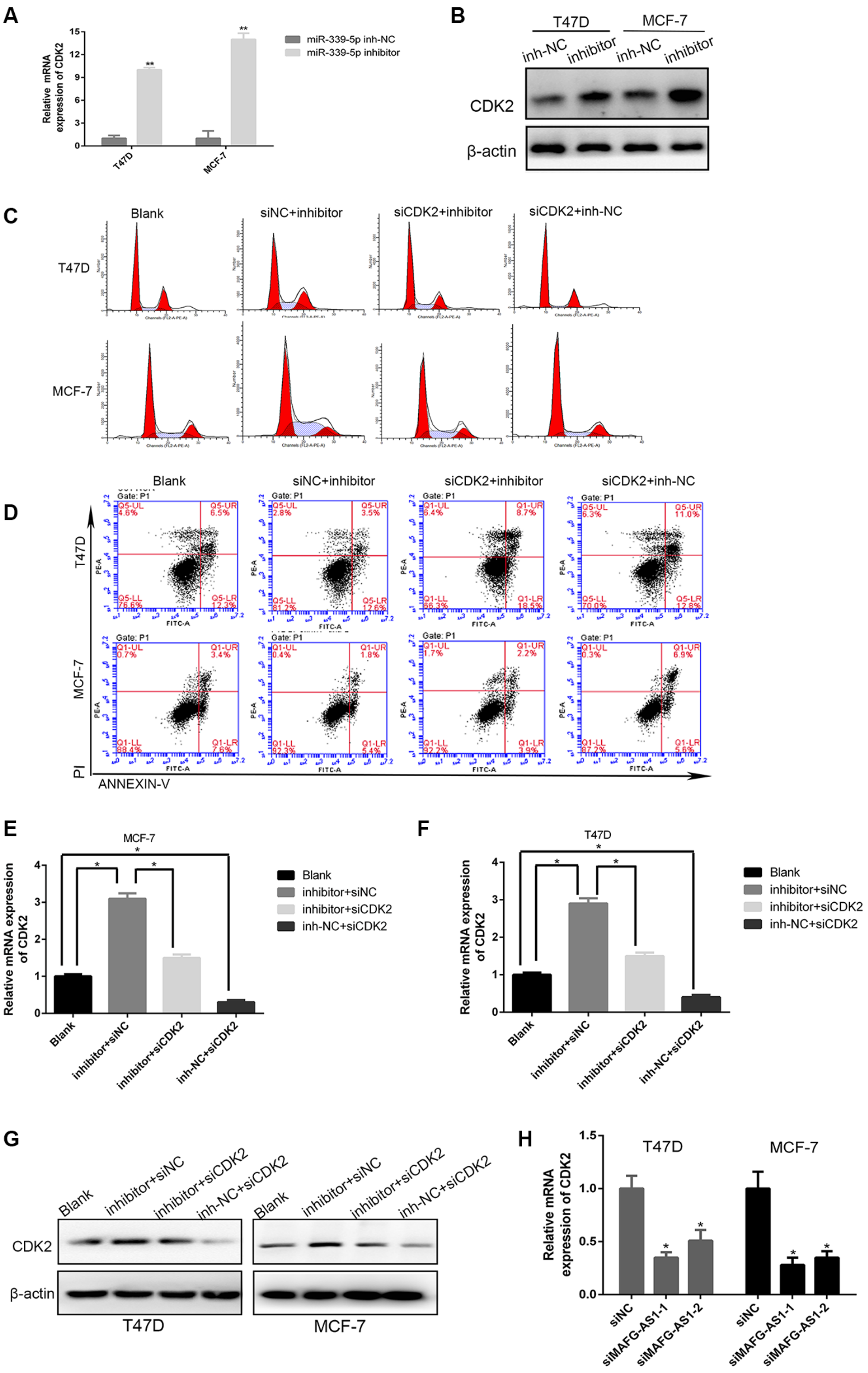

Supplementary Figure 4. Establishment of the MAFG-AS1/miR-339-5p/CDK2 axis. (**A**, **B**) The mRNA and protein levels of CDK2 in T47D and MCF-7 cells after being transfected with miR-339-5p inhibitor or inh-NC. (**C**, **D**) Knockdown of CDK2 abolished the miR-339-5p inhibitor induced cell growth shown by cell cycle distribution and apoptosis of T47D and MCF-7 cells. (**E**, **F**) The mRNA expression of CDK2 after CDK2 and miR-339-5p knockdown in T47D and MCF-7 cells. (**G**) The protein expression of CDK2 after CDK2 and miR-339-5p knockdown in T47D and MCF-7 cells. (**H**) The mRNA expression of CDK2 after siMAFG-AS1-1, siMAFG-AS1-2 or NC-siRNA transfection in T47D and MCF-7 cells.

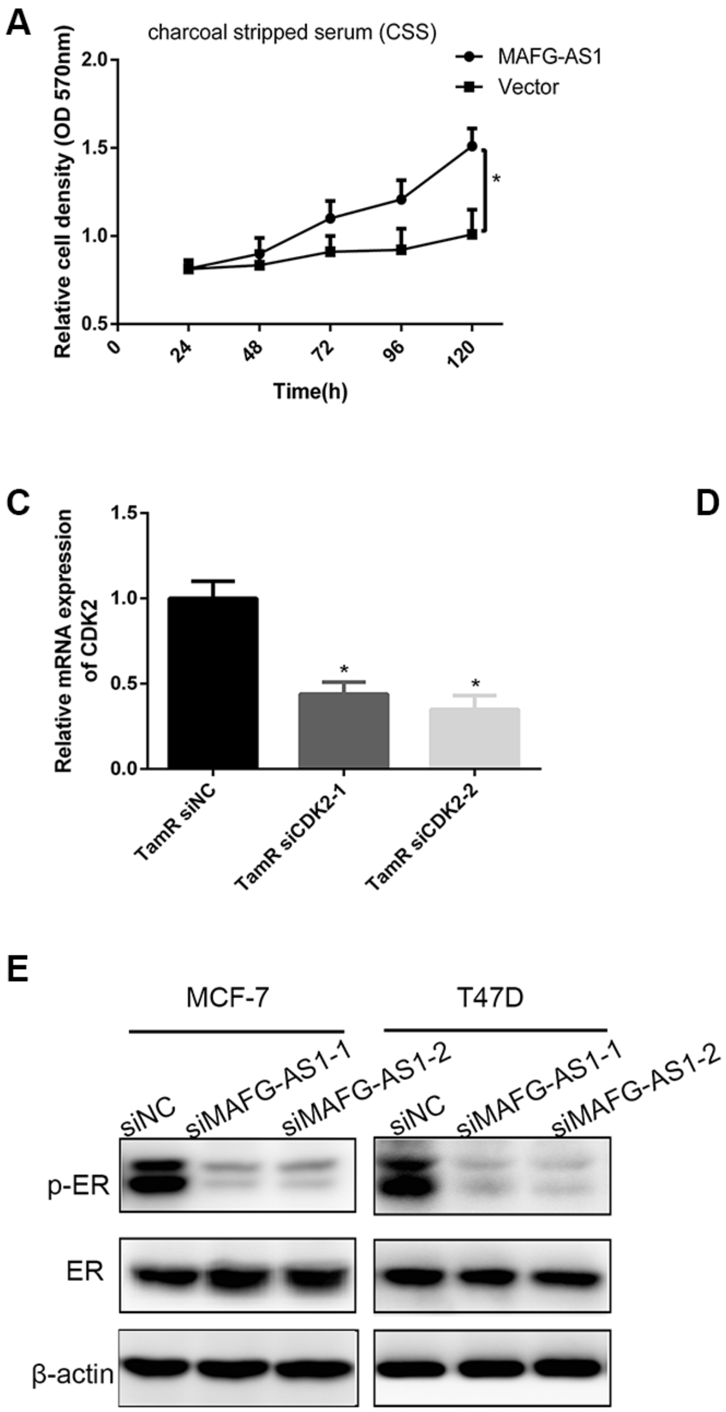

Supplementary Figure 5. Positive feedback loop between MAFG-AS1 and ERα. (**A**) Proliferation assay of T47D cells overexpressing MAFG-AS1 versus vector control in the presence of charcoal stripped serum (CSS), *p<0.05. (**B**) The mRNA expression of MAFG-AS1 in TamR MCF7 cells following siRNA knockdown of MAFG-AS1. Expression plotted relative to non-targeting siRNA control. (**C**) The mRNA expression of CDK2 in TamR MCF-7 cells following siRNA knockdown of CDK2. (**D**) qRT-PCR analysis of the expression of ESR1 compared siMAFG-AS1 with siNC in T47D and MCF-7 cells. (**E**) Western blot was performed to detect ER and p-ER expression compared si MAFG-AS1 with si NC in MCF-7 and T47D cells.
