# Supplementary material for: Cross-talk between the ER pathway and the lncRNA MAFG-AS1/miR-339-5p/ CDK2 axis promotes progression of ER+ breast cancer and confers tamoxifen resistance
Source: Aging (Albany NY). 2020 Oct 24;12(20):20658–83. doi: 10.18632/aging.103966 (PMC7655217; doi:10.18632/aging.103966)
Supplement: Supplementary Table 4 and 5 [file aging-12-103966-s005..pdf]

SUPPLEMENTARY TABLES

Supplementary Table 4. Primer sequence used in this study.

Name	Direction	Primer(5'-3')
MAFG-AS1	Forward	5'-CGGGAGGAAGATAAACGGGG-3'
	Reverse	5'-TGACCACGGAACACCTTCAG-3'
miR-339-5p	Forward	5’-GGGTCCCTGTCCTCCA-3’
	Reverse	5'-GCCTGAGATGAAGCACGTG-3'
CDK2	Forward	5′-CCAGGAGTTACTTCTATGCCTGA-3′
	Reverse	5′-TTCATCCAGGGGAGGTACAAC-3′
ESR1	Forward	5’-CCGGCTCCGCAAATGCTACGA-3’
	Reverse	5’-AGCGGGCTTGGCCAAAGGTT-3’
U6	Forward	5′-GCTTCGGCAGCACATATACTAAAAT-3′
	Reverse	5′-CGCTTCACGAATTTGCGTGTCAT-3′
18S	Forward	5′-CCCGGGGAGGTAGTGACGAAAAAT-3′
	Reverse	5′-CGCCCGCCCGCTCCCAAGAT-3′

Supplementary Table 5. siRNA sequences used in this study.

Name	Sequences (5'-3')
si-MAFG-AS1-1	5’-UCCUGAGAGCAGCAGAUCUTT-3’
si-MAFG-AS1-2	5’-GGAGUCAGGGCAAUUCCAATT-3’
si-MAFG-AS1-3	5’-GGUAACAUAGAGACCCUAUTT-3’
si-ESR1-1	5’-GGAGAAUGUUGAAACACAATT-3’
si-ESR1-2	5’-GGAUUUGACCCUCCAUGAUTT-3’
si-ESR1-3	5’-GGGCUCUACUUCAUCGCAUTT-3’
si-CDK2-1	5’-CGGAGCUUGUUAUCGCAAATT-3’
si-CDK2-2	5’-GAGUCCCUGUUCGUACUUATT-3’
si-CDK2-3	5’-CAAGAUCUCAAGAAAUUCATT-3’
si-NC	5’-AATTCTCCGAACGTGTCACGT-3’
